# Editorial: Current trends in endoscopic thoracic surgery: insights from the XXI SIET national meeting

**DOI:** 10.3389/fsurg.2023.1237928

**Published:** 2023-06-30

**Authors:** Federico Raveglia, Franca Melfi, Ugo Cioffi, Filippo Lococo, Sara Ricciardi, Cecilia Pompili, Giuseppe Cardillo

**Affiliations:** ^1^ Thoracic Surgery, IRCCS San Gerardo, Monza, Italy; ^2^ Thoracic Surgery, Robotic Multispecialty Centre for Surgery; Minimally Invasive and Robotic Thoracic Surgery; University Hospital of Pisa, Pisa, Italy; ^3^ Thoracic Surgery, University of Milan, Milano, Italy; ^4^ Thoracic Surgery, Fondazione Policlinico Universitario Agostino Gemelli IRCCS, Roma, Italy; ^5^ Thoracic Surgery, San Camillo Forlanini Hospital, Roma, Italy; ^6^ Thoracic Surgery, Leeds Institute of Cancer & Pathology-University of Leeds, Leeds, United Kingdom

**Keywords:** SIET, thoracic surgery, endoscopy, airways, national meeting, lung cancer, COVID-19

**Editorial on the Research Topic** Current trends in endoscopic thoracic surgery: insights from the XXI SIET national meeting

## Introduction

The Italian Society of Thoracic Endoscopy (SIET) was founded in 1980 to support research and innovation in endoscopic thoracic surgery by promoting its development and application nationally and internationally. SIET also promotes the training of young people through courses in collaboration with universities and scientific societies with similar aims. The SIET national meeting is tool for information and training exchange with the aim of creating a cohesive and active scientific community. The meeting takes place every two years and welcomes specialists in different disciplines (thoracic surgeons, pulmonologists, oncologists, radiologists). During the event, different topics are addressed among the most current in the field of minimally invasive thoracic surgery.

The 2022 XXI SIET national meeting was mainly focused on the management of lung and airways diseases with particular attention to the most challenging ongoing topics, each addressed by national and international experts presenting their personal experiences. COVID-19 related tracheal injuries, transition to RATS, innovative preoperative systemic therapies for lung cancer and awake surgery have been identified among the highly significant topics.

The goal of the current Research Topic was to promote the most interesting abstracts presented during the meeting, giving the opportunity to develop a full article manuscript.

## COVID-19 related tracheal injuries

The recent pandemic has worldwide impacted the healthcare system over the last 3 years, particularly involving pulmonologists and thoracic surgeons. An increasing number of patients have been subjected to prolonged invasive mechanical ventilation due to COVID-19 infection, leading to a significant number of post-intubation/tracheostomy upper airways lesions (stenosis or laceration) that needed endoscopic and/or surgical management (1).

In their review article Orlandi et al. focused on the different strategies to manage COVID-19-induced tracheal stenosis that may become a relevant pathology in the coming years. They conclude that this condition has distinctive features which differentiate it from other-causes stenosis and underline how the prognosis is related to (1) early diagnosis, (2) personalized and tailored treatments on each patient (through multidisciplinary discussion) (3) experienced tertiary centers referral. Therapeutic options consist of endoscopic or surgical procedures, which could provide high success and low complication rates when performed on selected patient at the right time.

Futhermore, Conforti et al. reported their meaningful experience with post intubation tracheal stenosis in COVID-19 patient with particular attention to relapses management. Their data confirm the prominent role of endoscopic therapy in the most of these challenging cases.

In turn, Passera et al. focused on post-intubation iatrogenic tracheobronchial injury (ITI) underlining that procedural and instrumental innovation, as well as medical development, have likely revolutionized the traditional management of post-intubation ITIs, broadening the use of conservative treatment and introducing the opportunity of the endoscopic approach, with interesting success and acceptable complications rates. Authors suggested the adoption of the risk-stratified morphological classification as that proposed by Cardillo et al. Cardillo et al. in particular, based on the personal experience with 62 patients affected by post-intubation tracheal laceration has confirmed the validation of the morphological classification, yet established by his team, as the major tool for defining the type of treatment. In addition, Brascia et al. presented their solid experience in the management of both tracheal stenosis and tracheal iatrogenic injuries COVID-19 related. This paper is very interesting since show the role of combined endoscopy and surgery in the most severe cases.

To conclude Tombelli et al. presented an innovative technique for tracheal laceration repairing with a hybrid mini-cervicotomic/endoscopic approach.

## Transition to RATS

In the last decade robot-assisted thoracoscopic surgery (RATS) has emerged as an alternative to video-assessed thoracoscopic surgery (VATS) for the treatment of lung cancer but concerns still exist regarding its learning curve and the high associated costs (2). Palleschi et al. reported their experience of transition from uniportal VATS to RATS with da Vinci Xi for lung resections. Their data investigated different variables such as number of nodes sampled, margins, conversion rate, complication and mortality rate, observing several practical advantages over VATS. In turn Harrison et al. presented a cost analysis of robotic vs. video-assisted thoracic surgery investigating the impact of the learning curve and the COVID-19 pandemic. Their results offered some evidence that the initial increased costs associated with RATS lung resection may be gradually offset as a program progresses.

## Awake surgery

Awake minimally invasive Uniportal Video Assisted Thoracic Surgery (U-VATS) has emerged as the last challenge in thoracic surgery that could change the future scenario for high comorbidity patients with early-stage lung cancer (3). Gonfiotti and co-workers presented a series of 10 high morbidity cases who underwent awake lung resection. Their results confirm recent literature data in favor of this technique and prompt further larger studies to reach stronger evidences to support it.

## Innovative preoperative systemic therapies for the lung

Nowadays, the role of immunotherapy and target therapy as induction or adjuvant therapy for resectable lung cancer in the set of a multimodal approach is the hottest topic in oncology thanks to recent trials encouraging results (4). Lampridis et al. have designed an updated overview of recent phase 3 randomized clinical trials on adjuvant and neoadjuvant immunotherapy or targeted therapy with an eye on some meaningful unresolved clinical issues, such as optimal duration of treatment, scheduling with respect to surgery and potential combinations of different systemic therapies.

Alongside these main topics, other emerging topics have been addressed by our experts in the field such as the surgical management of compensatory sweating after sympathectomy in hyperhidrosis or the role of surgery and medical approach in catamenial pneumothorax.

To conclude, during the 2022 SIET annual meeting thoracic surgeons and pulmonologists have discussed and reported their experience concerning advanced in thoracic pathologies with particular attention on new techniques and their impact on clinical practice. A picture has emerged showing that a continuous innovation in the field of surgery and interventional endoscopy is characterizing our days. Our research topic represents an insight in the hottest ongoing topics based on new data from the maximal experts in the field and an accurate literature review.
